# Neuropilin-1 facilitates SARS-CoV-2 cell entry and infectivity

**DOI:** 10.1126/science.abd2985

**Published:** 2020-10-20

**Authors:** Ludovico Cantuti-Castelvetri, Ravi Ojha, Liliana D. Pedro, Minou Djannatian, Jonas Franz, Suvi Kuivanen, Franziska van der Meer, Katri Kallio, Tuğberk Kaya, Maria Anastasina, Teemu Smura, Lev Levanov, Leonora Szirovicza, Allan Tobi, Hannimari Kallio-Kokko, Pamela Österlund, Merja Joensuu, Frédéric A. Meunier, Sarah J. Butcher, Martin Sebastian Winkler, Brit Mollenhauer, Ari Helenius, Ozgun Gokce, Tambet Teesalu, Jussi Hepojoki, Olli Vapalahti, Christine Stadelmann, Giuseppe Balistreri, Mikael Simons

**Affiliations:** 1 Institute of Neuronal Cell Biology, Technical University Munich, Munich, Germany.; 2 German Center for Neurodegenerative Diseases (DZNE), Munich, Germany.; 3 Faculty of Biological and Environmental Sciences, Molecular and Integrative Biosciences Research Program, University of Helsinki, Helsinki, Finland.; 4 Department of Neuropathology, University Medical Center Göttingen, Göttingen, Germany.; 5 Campus Institute for Dynamics of Biological Networks, University of Göttingen, Göttingen, Germany.; 6 Max Planck Institute for Experimental Medicine, Göttingen, Germany.; 7 Department of Virology, Medicum, University of Helsinki, Helsinki, Finland.; 8 Institute for Stroke and Dementia Research (ISD), University Hospital, LMU Munich, Munich, Germany.; 9 Helsinki Institute of Life Sciences–Institute of Biotechnology, University of Helsinki, Helsinki, Finland.; 10 Laboratory of Cancer Biology, Institute of Biomedicine and Translational Medicine, University of Tartu, Tartu, Estonia.; 11 Department of Virology, University of Helsinki and Helsinki University Hospital, Helsinki, Finland.; 12 Department of Health Security, Finnish Institute for Health and Welfare (THL), Helsinki, Finland.; 13 Clem Jones Centre for Ageing Dementia Research, Queensland Brain Institute, The University of Queensland, Brisbane, Queensland, Australia.; 14 Department of Anesthesiology and Intensive Care Medicine, University Medical Center Göttingen, Göttingen, Germany.; 15 Department of Neurology, University Medical Center Göttingen, Göttingen, Germany.; 16 Paracelsus-Elena-Klinik Kassel, Kassel, Germany.; 17 Institute of Biochemistry, ETH Zürich, Zürich, Switzerland.; 18 The Queensland Brain Institute, The University of Queensland, Brisbane, Queensland, Australia.; 19 Cancer Research Center, Sanford Burnham Prebys Medical Discovery Institute, La Jolla, CA, USA.; 20 Center for Nanomedicine and Department of Molecular, Cellular, and Developmental Biology, University of California, Santa Barbara, Santa Barbara, CA, USA.; 21 Institute of Veterinary Pathology, Vetsuisse Faculty, University of Zürich, Zürich, Switzerland.; 22 Department of Veterinary Biosciences, University of Helsinki, Helsinki, Finland.; 23 Munich Cluster of Systems Neurology (SyNergy), Munich, Germany.

## Abstract

The causative agent of coronavirus disease 2019 (COVID-19) is the severe acute respiratory syndrome coronavirus 2 (SARS-CoV-2). For many viruses, tissue tropism is determined by the availability of virus receptors and entry cofactors on the surface of host cells. In this study, we found that neuropilin-1 (NRP1), known to bind furin-cleaved substrates, significantly potentiates SARS-CoV-2 infectivity, an effect blocked by a monoclonal blocking antibody against NRP1. A SARS-CoV-2 mutant with an altered furin cleavage site did not depend on NRP1 for infectivity. Pathological analysis of olfactory epithelium obtained from human COVID-19 autopsies revealed that SARS-CoV-2 infected NRP1-positive cells facing the nasal cavity. Our data provide insight into SARS-CoV-2 cell infectivity and define a potential target for antiviral intervention.

## Another host factor for SARS-CoV-2

Virus-host interactions determine cellular entry and spreading in tissues. Severe acute respiratory syndrome coronavirus 2 (SARS-CoV-2) and the earlier SARS-CoV use angiotensin-converting enzyme 2 (ACE2) as a receptor; however, their tissue tropism differs, raising the possibility that additional host factors are involved. The spike protein of SARS-CoV-2 contains a cleavage site for the protease furin that is absent from SARS-CoV (see the Perspective by Kielian). Cantuti-Castelvetri *et al.* now show that neuropilin-1 (NRP1), which is known to bind furin-cleaved substrates, potentiates SARS-CoV-2 infectivity. NRP1 is abundantly expressed in the respiratory and olfactory epithelium, with highest expression in endothelial and epithelial cells. Daly *et al.* found that the furin-cleaved S1 fragment of the spike protein binds directly to cell surface NRP1 and blocking this interaction with a small-molecule inhibitor or monoclonal antibodies reduced viral infection in cell culture. Understanding the role of NRP1 in SARS-CoV-2 infection may suggest potential targets for future antiviral therapeutics.

*Science*, this issue p. 856, p. 861; see also p. 765

## Teaser

NRP1 serves as a host factor for SARS-CoV-2 infection and may potentially provide a therapeutic target for COVID-19.

An outbreak of severe acute respiratory syndrome coronavirus 2 (SARS-CoV-2) infections has caused a pandemic associated with a severe acute pulmonary disease named COVID-19 (coronavirus disease 2019) (*1*). A related coronavirus, SARS-CoV, led to a much smaller outbreak in 2003, possibly due to infection occurring predominantly in the lower respiratory system, whereas SARS-CoV-2 spreads rapidly through active pharyngeal viral shedding (*2*). Despite these differences, uptake of both viruses is mediated by the same cellular receptor: angiotensin-converting enzyme 2 (ACE2) (*3*–*5*). One hypothesis to explain the enhanced spreading of SARS-CoV-2 is the presence of a polybasic furin-type cleavage site, RRAR^S, at the S1-S2 junction in the SARS-CoV-2 spike (S) protein that is absent in SARS-CoV (*6*). Similar sequences are found in the S proteins of many other pathogenic human viruses, including Ebola, HIV-1, and highly virulent strains of avian influenza (*6*, *7*). The presence of the polybasic cleavage site in SARS-CoV-2 results in enhanced pathogenicity by priming the fusion activity (*8*) and could potentially create additional cell surface receptor binding sites. Proteolytic cleavage of RRAR^S by furin exposes a conserved C-terminal motif, RXXR_OH_ [where R is arginine and X is any amino acid; R can be substituted by lysine (K)], in the S protein. Such C-terminal sequences that conform to the C-end rule (CendR) are known to bind to and activate neuropilin (NRP1 and NRP2) receptors at the cell surface (*9*). Recent cryo–electron microscopy structures of the SARS-CoV-2 S protein demonstrated that the S1-S2 junction is part of a solvent-exposed loop and is therefore accessible for receptor interactions (*10*, *11*).

**Fig. 1:**
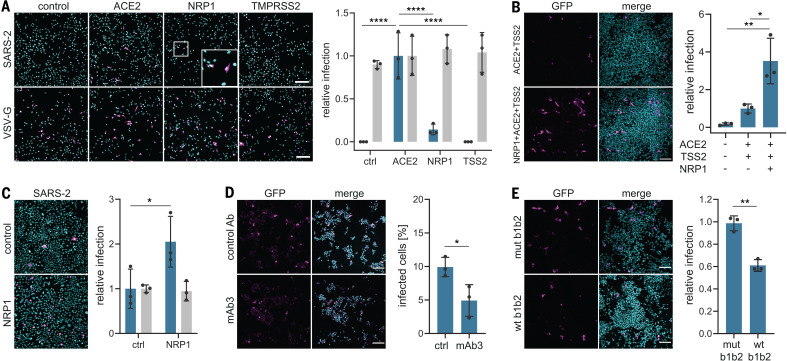
NRP1 facilitates the cellular entry of SARS-CoV-2 pseudotyped particles. (**A**) Representative images and quantification of SARS-CoV-2 S protein (SARS-2) (blue bars) and vesicular stomatitis virus glycoprotein (VSV-G) pseudotype (gray bars) infectivity in HEK-293T cells transiently expressing control (ctrl) vector, ACE2, NRP1, or TMPRSS2 (TSS2). Data are normalized to the respective infectivity of SARS-2 and VSV-G pseudotype in ACE2-expressing cells. Two-way analysis of variance (ANOVA) was carried out with Tukey’s correction for multiple comparisons. (**B**) HEK-293T cells transiently expressing ACE2 and TMPRSS2 or NRP1, ACE2, and TMPRSS2 were inoculated with SARS-2 pseudotype. Data are normalized to SARS-2 infectivity in cells expressing ACE2 and TMPRSS2. One-way ANOVA was performed with Tukey’s correction for multiple comparisons. (**C**) SARS-2 pseudotype infectivity in Caco-2 cells expressing NRP1 or control vector. Data are normalized to the respective infectivity of SARS-2 and VSV-G pseudotype in control cells. Two-way ANOVA was carried out with Sidak’s correction for multiple comparisons. (**D** and **E**) HEK-293T cells transiently expressing NRP1, ACE2, and TMPRSS2 were inoculated with SARS-2 pseudotype in the presence of mAb3 antibody against NRP1 [(D), mAb3] or control mAb2 [(D), ctrl Ab] and in the presence of soluble NRP1 wild-type b1b2 domain [(E), wt b1b2] or NRP1 mutant b1b2 domain [(E), mut b1b2]. Data in (E) are normalized to untreated cells expressing NRP1, ACE2, and TMPRSS2. Two-tailed unpaired Student’s *t* test was performed. All data are represented as means ± SDs from three independent experiments [(A) to (C)] or three biological replicates [(D) and (E)]. *P < 0.05, **P < 0.01, ****P < 0.0001. All images show GFP-positive, infected cells (magenta) and Hoechst stain (cyan). Scale bars, 100 μm.

To determine whether SARS-CoV-2 can use NRP1 for virus entry and infectivity, we generated lentiviral particles pseudotyped with the SARS-CoV-2 S protein. Pseudoviruses are well suited for virus entry assays, as they allow viral entry to be distinguished from other virus life-cycle steps. Human embryonic kidney 293 T (HEK-293T) cells, which have almost no detectable *ACE2* and *NRP1* transcripts (fig. S1), were transfected with plasmids that encode the two established host factors (*4*), human ACE2 and the transmembrane protease serine 2 (TMPRSS2), or NRP1. When expressed alone, ACE2 rendered cells susceptible to infection (Fig. 1A). Although NRP1 did not promote infection in HEK-293T cells, its coexpression with ACE2 and TMPRSS2 markedly enhanced infection (Fig. 1, A and B). NRP1 expression increased infection in Caco-2 cells, which endogenously express ACE2 (*12*) (Fig. 1C and fig. S1D), showing that NRP1 can potentiate infection in the presence of other host factors. To test the specificity of NRP1-dependent virus entry, we developed monoclonal antibodies (mAbs) that were designed to functionally block the extracellular b1b2 domain of NRP1, which is known to mediate binding to CendR peptides (*13*). The mAb3 was observed to bind to the recombinant b1b2 domain of wild-type (WT) NRP1 but not to the triple-mutant b1b2 domain (S346A, E348A, and T349A in the CendR binding pocket) (fig. S2A). The potency of the mAbs in preventing cellular binding and internalization of NRP ligands was tested using 80-nm silver nanoparticles (AgNP) coated with the prototypic NRP1-binding CendR peptide RPARPAR_OH_ (*9*) (fig. S2B). mAb3 efficiently blocked AgNP-CendR binding (fig. S2C) and internalization (fig. S2, D and E), whereas another monoclonal antibody, mAb2, had no effect and was used as a control in further experiments. Treatment of HEK-293T with mAb3 significantly reduced infection by SARS-CoV-2 pseudoviruses in cells expressing ACE2, TMPRSS2, and NRP1 (Fig. 1D), but not in cells expressing ACE2 and TMPRSS2 only (fig. S2F). When SARS-CoV-2 pseudovirus was preincubated with recombinant, soluble extracellular b1b2 domain of NRP1, the wild type significantly reduced infection compared with the triple mutant (Fig. 1E and fig. S2G).

**Fig. 2:**
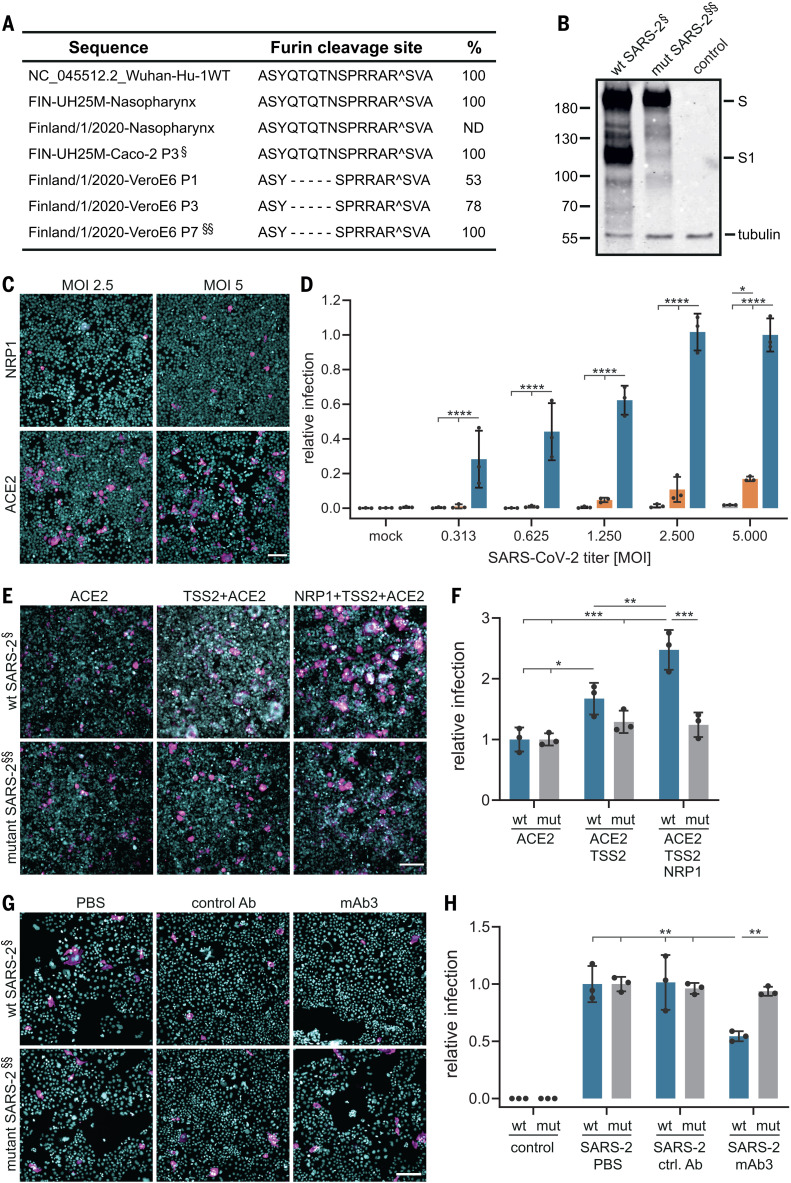
A blocking antibody against the b1b2 domain of NRP1 reduces infection by wild-type SARS-CoV-2 (SARS-2-wt) but not a mutant with a deletion at the furin-cleavage site (SARS-2-mut). (**A**) Sequence analysis of viruses isolated at different passages (P) from different cell types. The first sequence is the reference from the Wuhan isolate (NC_045512.2). The sequence abundance in each virus population is indicated as a percentage. ND, not determined. §, SARS-2-wt; §§, SARS-2-mut. A, Ala; S, Ser; Y, Tyr; Q, Gln; T, Thr; N, Asn; P, Pro; R, Arg; V, Val. (**B**) A deletion adjacent to the furin-cleavage site abrogates the enzymatic cleavage of the S protein. Immunoblot analysis was carried out on cell lysates from Vero-E6 cells infected for 16 hours with two viral populations (§ and §§). Numbers indicate protein size (in kilodaltons). (**C** and **D**) Representative images and quantification of SARS-2-wt infectivity in HEK-293T cells stably expressing ACE2 (blue bars) or NRP1 (orange bars) compared with nontransfected cells (gray bars). Different virus titers were used. Data are normalized to the infectivity in ACE2-expressing cells at multiplicity of infection (MOI) = 5. Two-way ANOVA was done with Tukey’s correction for multiple comparisons. (**E** and **F**) Representative images (E) and quantification (F) of HEK-293T cells stably expressing the indicated combinations of ACE2, TMPRSS2 (TSS2), and NRP1 after inoculation with SARS-2-wt (wt; blue bars) or SARS-2-mut (mut; gray bars). Data are normalized to the respective infectivity in ACE2-expressing cells. Two-way ANOVA was performed with Tukey’s correction for multiple comparisons. (**G** and **H**) Caco-2 cell infection in the presence of control mAb2 (ctrl. Ab) or mAb3 blocking antibodies against NRP1 after SARS-2-wt (wt; blue bars) or SARS-2-mut (mut; gray bars) inoculation. Data are normalized to the respective vehicle control (phosphate-buffered saline) sample. Two-way ANOVA was performed with Tukey’s correction for multiple comparisons. Data are means ± SDs from three independent experiments. *P < 0.05, **P < 0.01, ***P < 0.001, ****P < 0.0001. Magenta, SARS-2-wt– and SARS-2-mut–infected cells; Hoechst stain, cyan. Scale bars, 50 μm.

Next, we explored the role of NRP1 using SARS-CoV-2 isolated from COVID-19 patients from the Helsinki University Hospital. We used WT SARS-CoV-2 and a cleavage-impaired SARS-CoV-2 mutant that was isolated from Vero-E6 cells, which rapidly accumulate mutations at the furin cleavage site of the S protein during passaging (Fig. 2, A and B) (*14*). First, we confirmed that furin cleaved the WT, but not the mutant, SARS-CoV-2 S protein by analyzing S protein processing in Chinese hamster ovary cells with functional (parental) or deficient (FD11) furin enzyme (fig. S3) (*15*). Next, we validated that exogenous ACE2 expression rendered HEK-293T cells susceptible to infection with SARS-CoV-2 (Fig. 2, C and D). NRP1 expression alone caused lower levels of infection, which were detectable only with increasing virus titer (Fig. 2, C and D). We then compared the ability of WT and mutant SARS-CoV-2 to infect HEK-293T that stably express ACE2; ACE2 and TMPRSS2; or ACE2, TMPRSS2, and NRP1. Infection of these cell lines by the WT, but not the mutant, virus increased in the presence of NRP1, providing further evidence that NRP1 requires a furin-cleaved substrate for its effects (Fig. 2, E and F). We studied the effect of the NRP1-blocking antibody, mAb3, on infection of Caco-2 cells by WT and mutant SARS-CoV-2 and found that preincubation with NRP1-blocking antibody reduced WT virus infection by ~40%, whereas the control mAb2 had no effect (Fig. 2, G and H). NRP1-blocking antibody had no effect on the infection by the mutated virus (Fig. 2, G and H).

**Fig. 3:**
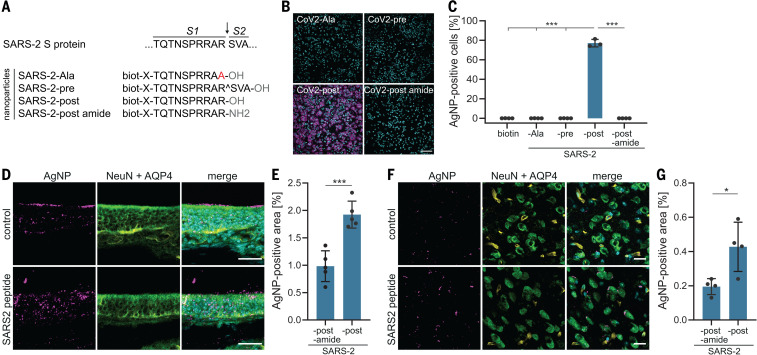
NRP mediates entry of nanoparticles coated with SARS-CoV-2 (SARS-2) S–derived CendR peptides into cultured cells, olfactory epithelium, and the central nervous system of mice. (**A**) Peptide sequences used for AgNP coating. The peptides mimic SARS-2 S protein after furin cleavage (post) and as controls; S protein before cleavage (pre), in which the terminal amino acid is replaced by alanine (Ala); or with an amide terminus (post amide). X, any amino acid. The arrow indicates the cleavage site. (**B** and **C**) Representative images and quantification of the internalization of peptide-coated AgNPs in HEK-293T cells expressing NRP1. Merged images show AgNP-positive cells (magenta) and Hoechst stain (cyan). One-way ANOVA was carried out with Tukey’s correction for multiple comparisons. (**D** to **G**) Representative images and quantification of the main olfactory epithelium [(D) and (E), respectively] and cortex [(F) and (G), respectively] 6 hours after intranasal administration of AgNPs coated with SARS2-post and SARS2-post amide peptides. *n* = 4 replicates for (C); *n* = 5 (E) and *n* = 4 (G) mice per condition. Data are means ± SDs. Two-tailed unpaired Student’s *t* test; *P < 0.05, ***P < 0.001. Magenta, AgNPs; cyan, Hoechst stain; green, NeuN (neuronal marker); yellow, AQP4. Scale bars, 100 μm (B), 20 μm [(D) and (F)].

Cleavage of SARS-CoV-2 S protein at the S1-S2 site generates the C-terminal end sequence TQTNSPRRAR_OH_. To determine whether this specific sequence can function as a substrate for NRP1, we used AgNPs coated with TQTNSPRRAR_OH_ peptide or different control peptides, including one with a terminal amide group (TQTNSPRRAR_NH2_), which reduces NRP1 binding (*9*) (Fig. 3A). We found that AgNP-TQTNSPRRAR_OH_, but not control AgNPs, were efficiently taken up by HEK-293T cells expressing NRP1 (Fig. 3, B and C). Next, we determined whether AgNP-TQTNSPRRAR_OH_ particles were also internalized into cells in vivo. We chose to study nanoparticle entry in the mouse olfactory epithelium, owing to the known expression of NRP1 in the olfactory system (*16*), including olfactory neuronal cells of the epithelium (fig. S4). AgNPs-TQTNSPRRAR_OH_ and control AgNP-TQTNSPRRAR_NH2_ were administered into the nose of anesthetized adult mice. Six hours after administration, we observed a significantly larger uptake of AgNP-TQTNSPRRAR_OH_ than of AgNP-TQTNSPRRAR_NH2_ into the olfactory epithelium (Fig. 3, D and E) and, unexpectedly, into neurons and blood vessels of the cortex (Fig. 3, F and G). Similar results were obtained for AgNPs coated with the prototypic NRP1-binding CendR peptide RPARPAR_OH_ (fig. S5).

Having obtained evidence for a role of NRP1 in cell entry of SARS-CoV-2, we examined whether NRP1 expression correlated with the detection of virus RNA in single-cell transcriptomes. For these analyses, we used published single-cell RNA sequencing (scRNA-seq) datasets of cultured experimentally infected human bronchial epithelial cells and cells isolated from bronchoalveolar lavage fluid (BALF) of severely affected COVID-19 patients (*17*). Among the proposed entry and amplification factors, *NRP1*, *FURIN*, and *TMPRSS11A* were enriched in SARS-CoV-2–infected cells compared with noninfected cells (fig. S6). We also detected increased expression of these proteins after infection (fig. S6). In addition, RNA expression of *NRP1* and its homolog *NRP2* was elevated in SARS-CoV-2–positive cells compared with adjacent cells in the BALF of severely affected COVID-19 patients (fig. S7).

Because the availability of virus receptors and entry cofactors on the surface of host cells determines infectivity, we compared the expression patterns of *ACE2* and *NRP1* in published scRNA-seq datasets of human lung tissue (*18*) and human olfactory epithelium (*19*). Whereas *ACE2* was detected at very low levels, both *NRP1* and *NRP2* were abundantly expressed in almost all pulmonary and olfactory cells, with the highest levels of expression in endothelial cells (figs. S8 and S9). We confirmed these results by examining NRP1 immunoreactivity in human autopsy tissue and detected NRP1 in the epithelial surface layer of the human respiratory and olfactory epithelium (fig. S10A). ACE2 was hardly detectable in these tissues (fig. S10B). Within the olfactory epithelium, NRP1 was also observed in cells positive for oligodendrocyte transcription factor 2 (OLIG2), which is mostly expressed by olfactory neuronal progenitors (fig. S10C).

**Fig. 4:**
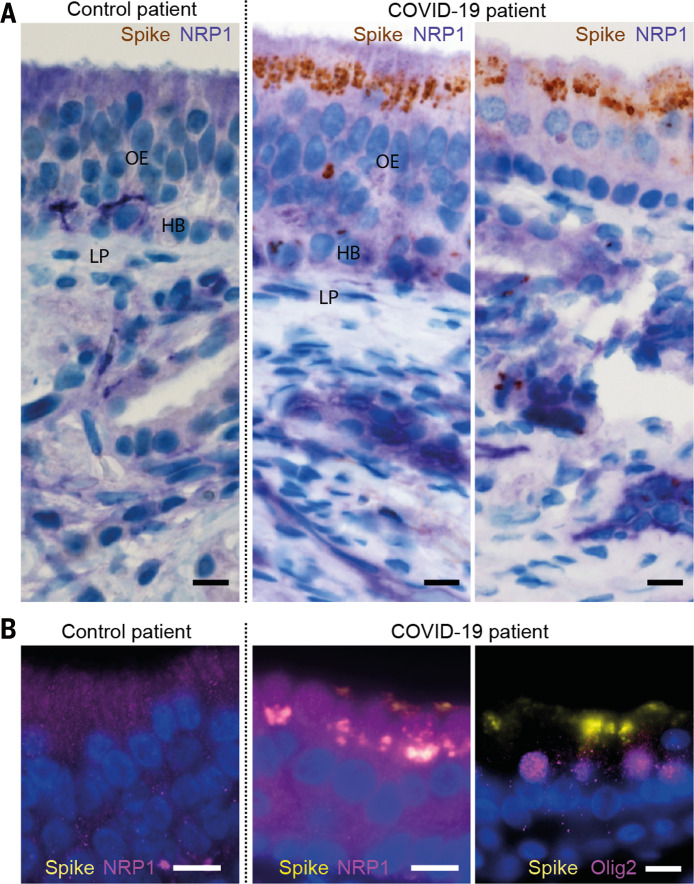
SARS-CoV-2 infects the olfactory epithelium. (**A**) Costaining of S protein (brown) and NRP1 (purple) in the apical olfactory epithelium (OE) in a noninfected control (left) and in the apical OE (middle) and adjacent mucosa (right) in a COVID-19 patient. LP, lamina propria; HB, horizontal basal cells. (**B**) Costaining of NRP1 (magenta) or OLIG-2 (magenta) with S protein (yellow) in OE cells. Nuclei are shown in blue. Scale bars, 10 μm.

In light of the widely reported disturbance of olfaction in a large fraction of COVID-19 patients (*20*, *21*) and the enrichment of NRPs in the olfactory epithelium, we analyzed a series of autopsies from six COVID-19 patients and eight noninfected control patients to determine whether SARS-CoV-2 could infect NRP1-positive cells (Fig. 4 and table S1). Using antibodies against the S protein, we detected infection of the olfactory epithelium in five of six COVID-19 patients. The infected olfactory epithelial cells showed high expression of NRP1 (Fig. 4, A and B). Additional costaining indicated infection of cells positive for OLIG2 (Fig. 4B and fig. S11).

There is limited knowledge about the virus–host interactions that determine cellular entry of SARS-CoV-2. Viruses display considerable redundancy and flexibility because they can exploit weak multivalent interactions to enhance affinity. To date, studies of SARS-CoV-2 entry have focused almost entirely on ACE2, which is expressed at very low protein levels in respiratory and olfactory epithelial cells (*22*). This raises the possibility that cofactors are required to facilitate virus–host cell interactions in cells with low ACE2 expression. NRP1 could represent such an ACE2 potentiating factor by promoting the interaction of the virus with ACE2. The reason a number of viruses (*23*–*26*) use NRPs as entry factors may be their high expression on epithelia facing the external environment and their function in enabling cell, vascular, and tissue penetration (*9*, *13*).

## Notes

**Correction (12 November 2020):** After the First Release version was published, the authors added an image to Fig. 4A so that the figure now shows a region of the olfactory epithelium of a COVID-19 patient that is comparable to the region shown for the control patient.

## Supplementary Materials

science.sciencemag.org/content/370/6518/856/suppl/DC1 Materials and Methods Figs. S1 to S11 Table S1 References (*27*–*37*) MDAR Reproducibility Checklist

## Appendix

View/request a protocol for this paper from *Bio-protocol*.
